# Fibroblast senescence-associated extracellular matrix promotes heterogeneous lung niche

**DOI:** 10.1063/5.0204393

**Published:** 2024-06-05

**Authors:** Andrew M. Howes, Nova C. Dea, Deepraj Ghosh, Krishangi Krishna, Yihong Wang, Yanxi Li, Braxton Morrison, Kimani C. Toussaint, Michelle R. Dawson

**Affiliations:** 1 Department of Molecular Biology, Cell Biology, and Biochemistry, Brown University, Providence, Rhode Island 029012, USA; 2 School of Engineering, Brown University, Providence, Rhode Island 02912, USA; 3 Department of Pathology and Laboratory Medicine, Brown University, Providence, Rhode Island 02912, USA

## Abstract

Senescent cell accumulation in the pulmonary niche is associated with heightened susceptibility to age-related disease, tissue alterations, and ultimately a decline in lung function. Our current knowledge of senescent cell-extracellular matrix (ECM) dynamics is limited, and our understanding of how senescent cells influence spatial ECM architecture changes over time is incomplete. Herein is the design of an *in vitro* model of senescence-associated extracellular matrix (SA-ECM) remodeling using a senescent lung fibroblast-derived matrix that captures the spatiotemporal dynamics of an evolving senescent ECM architecture. Multiphoton second-harmonic generation microscopy was utilized to examine the spatial and temporal dynamics of fibroblast SA-ECM remodeling, which revealed a biphasic process that established a disordered and heterogeneous architecture. Additionally, we observed that inhibition of transforming growth factor-β signaling during SA-ECM remodeling led to improved local collagen fiber organization. Finally, we examined patient samples diagnosed with pulmonary fibrosis to further tie our results of the *in vitro* model to clinical outcomes. Moreover, we observed that the senescence marker p16 is correlated with local collagen fiber disorder. By elucidating the temporal dynamics of SA-ECM remodeling, we provide further insight on the role of senescent cells and their contributions to pathological ECM remodeling.

## INTRODUCTION

Aging of the lung is linked to the accumulation of senescent cells, increased chronic inflammation and subsequent modifications to the extracellular matrix (ECM).^1^ It has been established that this accumulation of senescent cells generates a higher risk for age-related diseases, such as cancer, cardiovascular disease, neurodegenerative disease, and fibrotic disease.^2–5^ Chronic respiratory diseases are currently the third leading cause of death in the US for those over 65 years of age.^6^ By 2050, it is estimated that 1 in 4 Americans will be above the age of 65, leading to an increase in the prevalence of age-related pulmonary disease.^7^ Therefore, it is of pertinence to study the influence of senescent cells on the architecture of the lung.

Cellular senescence is a prevailing aspect of pulmonary fibrosis, whereby senescent cells directly remodel ECM or indirectly through senescence-associated secretory phenotype (SASP) signaling to neighboring cells.^8,9^ This state of irreversible cell-cycle arrest is induced by pro-aging factors, like mitochondrial dysfunction, epigenetic alterations, telomere attrition, genomic instability, and loss of proteasome activity.^10^ Upon increase in any of these factors, pre-senescent cells exit the cell-cycle and become post-mitotic by the activation of the p53/p21^CIP1^ and p16^INK4a^/pRB pathways. After the induction of senescence, senescent cells then release SASP into the surrounding environment. SASP contains multiple factors capable of remodeling the ECM environment, such as matrix metalloproteinases (MMPs), collagen crosslinkers (LOX, TGM2), and pro-inflammatory regulators known to influence the matrix remodeling potential of surrounding cells (TGF-*β*, IL-6, and IL-8).^11–13^ The buildup of senescent cells consequently dysregulates tissue homeostasis, resulting in a heightened inflammatory state through the overexpression of ECM-degrading enzymes, chemokines, cytokines, and growth factors.^14,15^ Consequent chronic inflammation by apoptosis resistant senescent cells disrupts ECM remodeling homeostasis, resulting in a perpetual pro-fibrotic niche that leads to the loss of tissue function.^16^

Due to the pleiotropic nature of senescence, our understanding of how senescent fibroblasts remodel the ECM architecture organization over time is misunderstood. Muñoz-Espín *et al.* propose that transient cellular senescence promotes tissue remodeling in three sequential events: senescence, clearance, and regeneration. However, persistent damage over time compromises this model and the lack of senescent cell clearance and resolution results in senescent lesions.^17^ It is indicated that idiopathic pulmonary fibrosis (IPF), a chronic age-related pulmonary fibrotic disease, is influenced by cellular senescence.^18^ Furthermore, IPF fibroblasts have been shown to display senescence-like phenotypes.^9,19^ Herein, the disruption of immunosurveillance due to senescent cells themselves, or in part an aging immune system, results in the accumulation of senescent cells within the tissue niche.^20^ Moreover, TGF-β, a profibrotic cytokine known to orchestrate the development of IPF and other fibrotic diseases, is abundantly found in senescent cell SASP.^21–24^ Additionally, it has been shown to induce an immunosuppressive senescent cell state and induce cell homing defects.^25,26^ Although TGF-β is involved in a myriad of other signaling processes, its ability to stimulate profibrotic ECM remodeling is one of its hallmark traits. Excessive TGF-β-induced ECM remodeling ultimately leads to progressive fibrotic tissue-level alterations resulting in decreased lung function in both mice and humans.^27^

We expand upon the work done by Choi *et al.* by examining senescent ECM structural alterations that influence cell and tissue behavior over time.^28^ This study expands upon their work by quantifying the spatial and temporal dynamics of senescent pulmonary fibroblasts *in vitro* and their impact on the ECM architecture of the lung niche. Previous senescence models have not examined dynamic senescent cell changes of the senescent matrix and how senescent cells influence matrix architecture over time. Therefore, we designed an *in vitro* senescent lung fibroblast-derived matrix (FDM) model to study SA-ECM architecture changes and spatial remodeling during the induction of senescence. Using multiphoton second-harmonic generation (SHG) microscopy, we identified distinct structural biomarkers of an evolving pathological SA-ECM architecture undergoing phase-like changes to stabilize a heterogeneously disorganized senescent matrix over time. We hypothesized that senescent fibroblasts contributed to the disorganization of the matrix mechanically by having disordered actin cytoskeleton and an increased abundance of integrin binding compared to pre-senescent cells. Previously, it was not clear if senescent fibroblasts promote ECM disorder biophysically. Our findings indicate that senescent fibroblast actin cytoskeleton disorganization and elevated integrin adhesion result in multilateral tension on the matrix that promotes disorganized collagen fiber organization.

Based on the latter observation that disordered senescent fibroblast polarization and cytoskeleton tension are applied to the matrix in a multilateral fashion, we separately inhibited mechanoresponsive myocardin-related transcription factor-A (MRTF-A) and signaling of the TGF-*β* pathway through TGF*β*R1. The MRTF-SRF pathway is a critical regulator of cell-ECM dynamics that links actin cytoskeleton organization with transcriptional regulation, providing essential signals for adhesion, cell polarization, and contractility.^29^ Furthermore, mechanical stress due to stiffening of the ECM can modulate MRTF-A signaling, contributing to an α-smooth muscle actin (αSMA) stress-fiber expressing myofibroblast phenotype that is responsible for fibrotic remodeling.^30–32^ Moreover, elevated levels of *α*v (CD51) and *β*1 (CD29) integrins have been shown to increase the localization of MRTF-A to the nucleus, resulting in heightened activation and contractility.^33^ By inhibiting the nuclear localization of MRTF-A, we aimed to reduce SA-ECM disorganization by limiting senescent fibroblast multilateral tension on the matrix. Second, TGF-β is a well-known pro-fibrotic cytokine that influences ECM remodeling potential and is expressed within SASP. By blocking TGF-β signaling through TGFβR1, we intended to limit the senescent fibroblast capability to remodel a disorganized SA-ECM architecture. Inhibition of the TGF-*β* pathway resulted in the improvement of local SA-ECM architecture, generating more organized anisotropic collagen fibers compared to MRTF-A inhibition. Finally, we examined clinical patient samples to understand how collagen fiber organization changes with the age and the percent of p16 positive cells within patient tissue diagnosed with interstitial pulmonary fibrosis. Here, we show a linear relationship between the increasing age of pulmonary fibrosis patients and local collagen fiber disorganization. Moreover, we also observed a correlation between the percent of p16 positive cells and local collagen fiber disorder within these patient samples.

By spatiotemporally modeling the stabilization of a senescent matrix, our results further demonstrate that an abundance of senescent cells plays a potent role in the deregulation of tissue remodeling and exhibit temporally different ECM remodeling phases to establish a distinct heterogeneously disorganized pathological architecture over time. Additionally, our findings report an innovative *in vitro* senescent fibroblast-derived matrix model that captures the spatial and temporal dynamics of senescent fibroblast matrix remodeling. Utilizing multiphoton SHG microscopy, we establish that SA-ECM remodeling is temporally biphasic and establishes a heterogeneously disorganized ECM architecture. We further connect these results by showing a correlation between age, p16 positivity, and ECM disorganization within patients diagnosed with pulmonary fibrosis. These findings build upon previous observations that the senescent fibroblast phenotype promotes a pathological SA-ECM architecture and plays a pivotal role in promoting tissue dysfunction.

## RESULTS

### Primary lung fibroblasts acquire senescent phenotype

**FIG. 1.**
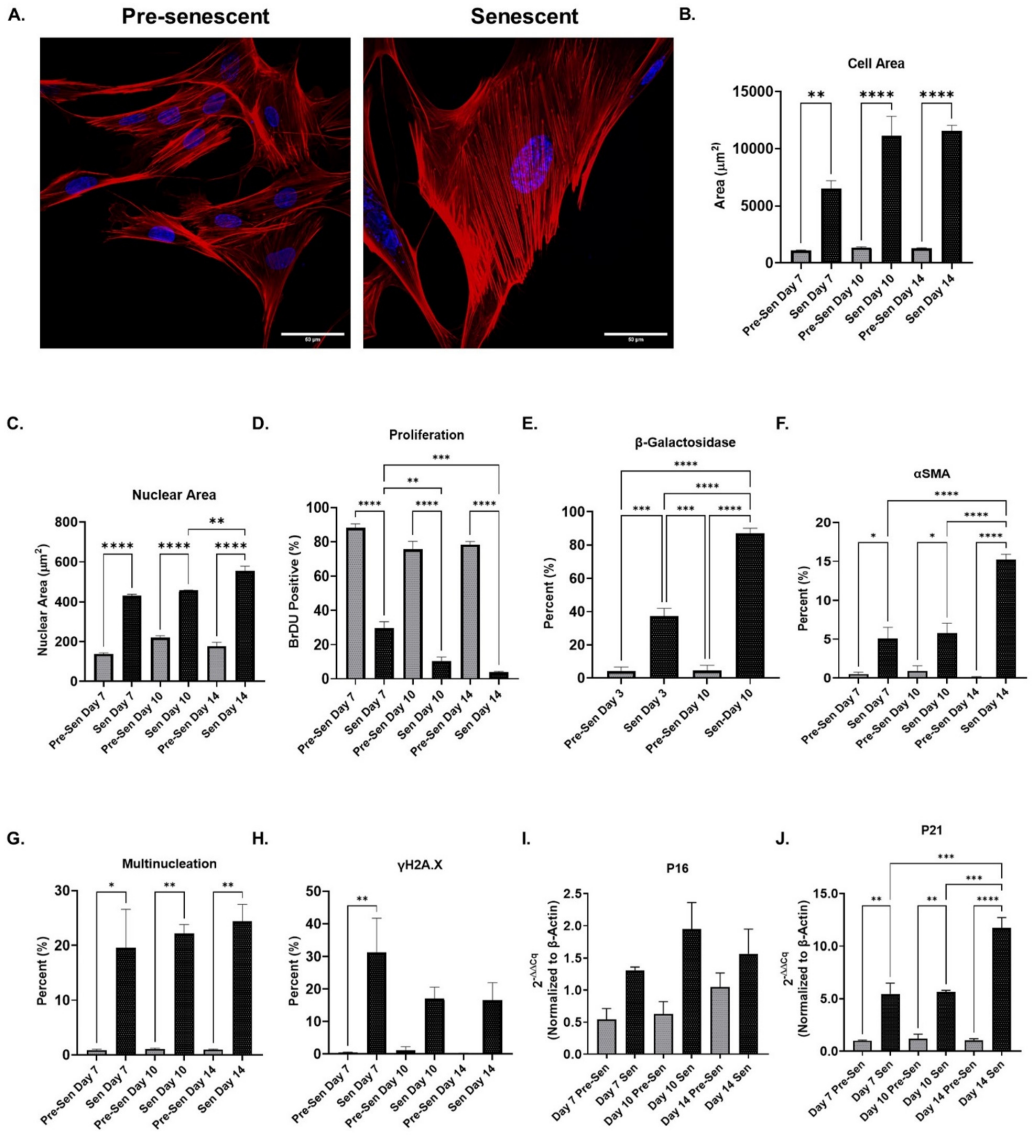
Primary lung fibroblasts acquire senescent phenotype. (a) Representative confocal microscopy images of pre-senescent and senescent lung fibroblasts stained with Hoechst 33342 and phalloidin rhodamine (obj. = 60×, N.A. = 1.3, scale bar = 50 *μ*m). (b) Nuclear area of pre-senescent and senescent lung fibroblasts over time (n ≥ 162, N = 3). (c) Cell area changes were tracked over time for pre-senescent and senescent lung fibroblasts (n ≥ 81, N = 3). (d) Bromodeoxyuridine (BrDU) labeling confirmed growth arrest of irradiated fibroblasts post-irradiation (n ≥ 151, N = 3). (e) The percent of SA-β-Gal expressing lung fibroblasts for pre-senescent and senescent fibroblasts (n ≥ 238, N = 3). (f) The percent of pre-senescent and senescent fibroblasts positive for αSMA stress-fibers (n ≥ 151, N = 3). (g) The percent of multinucleated pre-senescent and senescent fibroblasts over time (n ≥ 151, N = 3). (h) The percent of pre-senescent and senescent nuclei positive for γH2A.X over time (n ≥ 151, N = 3). (i) qRT-PCR analysis of p16 for pre-senescent and senescent fibroblasts over time (N =3). (j) qRT-PCR analysis of p21 for pre-senescent and senescent fibroblasts over time (N = 3). Mean ± SEM; ANOVA, ^*^P ≤ 0.05, ^**^P ≤ 0.01, ^***^P ≤ 0.001, ^****^P ≤ 0.0001.

To generate a population of primary senescent pulmonary fibroblasts, γ-irradiation was used to induce a stress-induced premature senescent (SIPS) phenotype. A concurrent analysis of the senescent cell phenotype was conducted, examining cell-cycle arrest, morphological changes, and DNA damage and senescence-associated β-galactosidase (SA-β-Gal) was used to validate fibroblast senescence.^34^ Irradiated fibroblasts increased in cellular and nuclear area over time compared to non-irradiated conditions (pre-senescent) [Figs. 1(a)–1(c)]. Cell-cycle arrest was measured using bromodeoxyuridine (BrDU), where at day 14 post-irradiation less than 4% of irradiated fibroblasts were positive for BrDU [Fig. 1(d)]. The presence of the senescence marker SA-β-Gal was measured post-irradiation and reached 90% positivity by day 10 [Fig. 1(e)]. Senescence and myofibroblast differentiation have previously been linked, showing that senescent cells heterogeneously express α-smooth muscle actin (αSMA) stress-fibers.^35^ Therefore, fluorescence microscopy was used to measure the percent of αSMA positive fibroblasts. The percent of irradiated fibroblasts positive for αSMA was higher than pre-senescent conditions over 14 days (5–15% vs 0.4–1% accordingly) [Fig. 1(f), see supplementary material Fig. 1(a)]. SIPS also resulted in an increase in multinucleation over time compared to pre-senescent conditions (25% vs ≤ 1%) [Fig. 1(g)]. DNA damage was observed via staining for DNA double-strand break marker γH2A.X. The percent of senescent fibroblasts positive for γH2A.X was significantly higher at day 7 (31% vs 0.4%) and remained increased compared to pre-senescent conditions [Fig. 1(h), see supplementary material Fig. 1(b)]. Finally, qRT-PCR was used to measure expression levels of cyclin-dependent kinase inhibitors p16 and p21. Senescent fibroblast p16 expression levels were not significantly different from those of pre-senescent fibroblasts [Fig. 1(i)]. However, p21 expression levels were significantly higher than that of pre-senescent fibroblasts [Fig. 1(j)].

### Generation of senescent primary lung fibroblast-derived matrix model

**FIG. 2.**
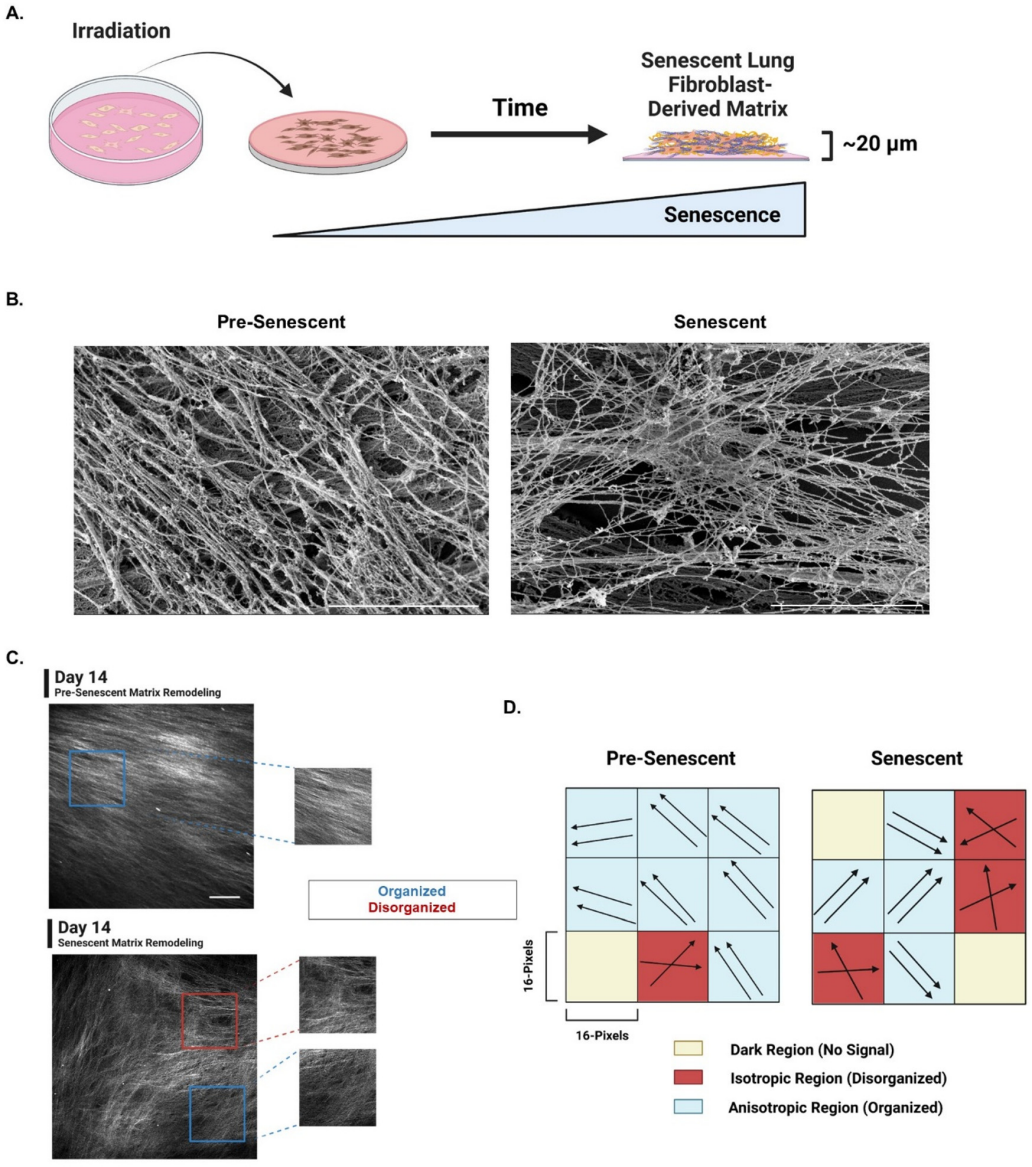
Generation of senescent primary lung fibroblast-derived matrix model. (a) Pre-senescent and senescent lung fibroblasts were used to generate fibroblast-derived matrices (FDMs) over time (Created with BioRender.com). (b) SEM images of decellularized pre-senescent and senescent lung fibroblast FDMs (scale bar = 5 *μ*m). (c) Representative second-harmonic generation (SHG) microscopy images of pre-senescent and senescent FDMs at day 14. Examples of organized (anisotropic) regions (blue) and disorganized (isotropic) regions (red) are denoted (obj. = 25×, N.A. = 1.05, scale bar = 100 *μ*m.) (d) Schematic depicting the SHG image regional analysis used to measure changing collagen fiber organizations and heterogeneity of pre-senescent and senescent FDMs. 16 × 16-pixel^2^ regions generated a grid array over SHG images and within each region the individual collagen fiber angles were measured. Regions were then labeled isotropic (disorganized; fibers do not have a preferred orientation), anisotropic (organized; fibers have a preferred orientation), and dark (no signal) (Created with BioRender.com).

To grow senescent ECM and quantify SA-ECM remodeling by senescent pulmonary fibroblasts, we designed a senescent FDM model. Pre-senescent and senescent lung fibroblasts were seeded to gelatin-coated coverslips and fed matrix promoting growth media to develop FDMs of thickness 20–30 *μ*m [Fig. 2(a)]. Scanning electron microscopy (SEM) of decellularized pre-senescent and senescent FDMs was used to qualitatively observe the ultrastructure of the pre-senescent and senescent extracellular matrix [Fig. 2(b)]. Multiphoton SHG microscopy was used to optically section the FDM architectures [Fig. 2(c)]. Using an in-house 2D fast Fourier transform (2D FFT) MATLAB code, individual SHG optical sections were overlaid with a grid consisting of 16 × 16 pixel regions (1048 total regions) to quantify local individual collagen fiber organizational changes within each 16 × 16 pixel region.^36–39^ Circular variance was used as a metric to quantify collagen fiber disorganization and ranged from 0 to 1. A circular variance of 0 on a polar coordinate system would result in a single line and parallel collagen fiber organization. On the contrary, a circular variance of 1 would result in a circle on a polar coordinate system and indicate complete collagen fiber disorganization. The circular variance of individual collagen fiber orientations identified within each region was used to classify the regions as isotropic (disorganized-fibers), anisotropic (organized-fibers), or dark (no signal). Global circular variance was used as a bulk metric that depicts the overall circular variance of collagen fiber orientations across the entire image and consisted of both anisotropic and isotropic regions. Anisotropic circular variance was used as a localized collagen fiber orientation metric that compared only anisotropic (organized) regions with a preferred orientation to one another. It is important to note that two anisotropic regions identified can have different orientations, as exampled in the cartoon depiction [Fig. 2(d)]. These metrics in culmination were used to quantitatively measure heterogeneous organizational changes of SA-ECM remodeling over time.

### Timeline of SA-ECM fiber density and orientation alterations

**FIG. 3.**
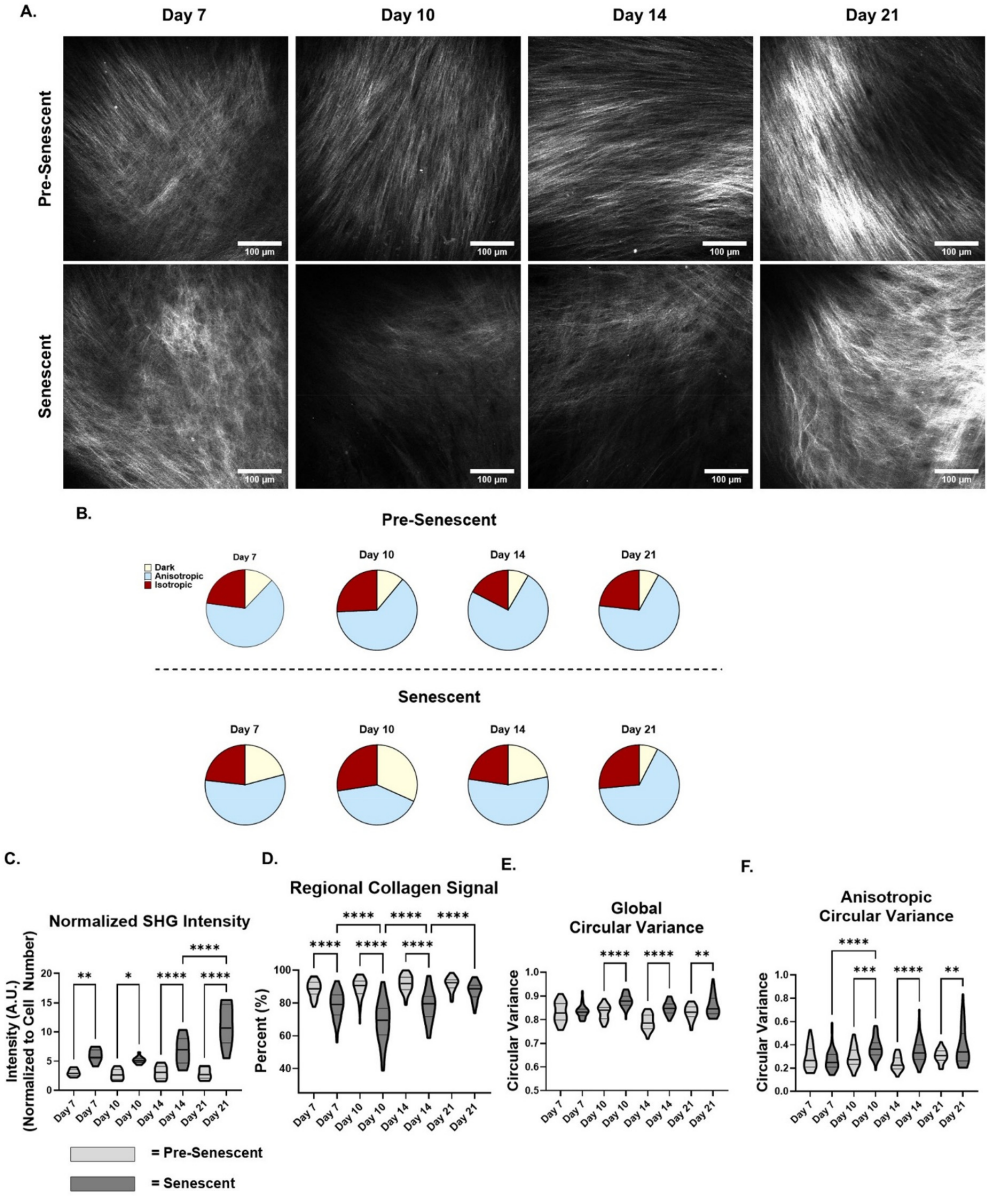
Timeline of SA-ECM fiber density and orientation alterations. (a) Representative multiphoton second-harmonic generation (SHG) images of pre-senescent and senescent fibroblast-derived matrices (FDMs) imaged at days 7, 10, 14, and 21 (obj. = 25×, N.A. = 1.05, scale bar = 100 *μ*m. (b) Distribution of anisotropic, isotropic, and dark regions classified over 21 days for pre-senescent and senescent FDMs (n ≥ 36, N = 3, N = 2 for day 21 senescent). (c) SHG intensity normalized to cell number per imaged region (n ≥ 8, N = 3, N = 2 for day 21 senescent). (d) Percent regional collagen signal of senescent and pre-senescent FDM (n ≥ 36, N = 3, N = 2 for day 21 senescent). (e) Global circular variance of individual collagen fibers (n ≥ 36, N = 3, N = 2 for day 21 senescent). (f) Anisotropic circular variance for pre-senescent and senescent FDMs (n ≥ 36, N = 3, N = 2 for day 21 senescent). ANOVA, ^*^P ≤ 0.05, ^**^P ≤ 0.01, ^***^P ≤ 0.001, ^****^P ≤ 0.0001.

Quantitative analysis of multiphoton SHG microscopy images was used to measure SA-ECM remodeling over time. We assessed collagen remodeling of pre-senescent and senescent FDMs on days 7, 10, 14, and 21 [Fig. 3(a)]. Of the regions identified through the grid analysis, pre-senescent FDMs had consistent percent of anisotropic regions throughout 21 days (63%–74%). However, senescent conditions decreased in the percent of anisotropic regions from 56% to 40% from days 7 to 10 and then increased to 66% by day 21 [Fig. 3(b)]. The percent of isotropic regions identified stayed relatively consistent for both pre-senescent and senescent conditions over the course of 21 days, only fluctuating 5% [Fig. 3(b)]. SHG intensity was quantified and normalized to cell number per region imaged, which served as a proxy for collagen deposition per cell [Fig. 3(c)]. Here, we observed that pre-senescent conditions had minimal changes in SHG intensity. Nevertheless, senescent FDMs showed a parabolic pattern in normalized SHG intensity by first decreasing and then significantly increasing [Fig. 3(c)]. To examine regional matrix density changes, the percent of regions containing matrix (anisotropic and isotropic regions) was quantified as regional collagen signal. We measured a similar parabolic trend in the percent of regions having collagen for senescent matrices. Between days 7 and 10, the percent of regions with collagen signal decreased from 79% to 68% for the senescent conditions (P < 0.0001). Interestingly, from days 10 to 14 we saw a recovery in the percent of regions containing collagen signal to 78% (P < 0.0001) and a further significant increase to similar levels as pre-senescent FDMs by day 21 (87% vs 91% accordingly, P = 0.0930) [Fig. 3(d)]. Even though senescent matrices recovered to similar levels of collagenous regions as pre-senescent conditions, circular variance analysis of collagen fibers revealed significant increases in matrix disorganization based on global and anisotropic circular variance after day 7 [Figs. 3(e) and 3(f)]. Consistent with previous reports, senescent fibroblasts had significantly higher proteolytic activity at day 10, which coincided with the decrease in the percent of collagenous regions and normalized SHG intensity [see supplementary material Fig. 2(a)].^13^ Like previous observations, qRT-PCR analysis of senescent fibroblasts revealed a significant decrease in COL1A1 expression and increased expression of COL3A1, TGM2, and LOX over 21 days^14,40^ [see supplementary material Figs. 2(b)–2(e)]. Interpreting the collective data, senescent cells establish a heterogeneously disorganized SA-ECM through multi-step remodeling, first degrading the ECM and then accumulating collagen that could be over-modified and unable to be degraded due to excessive cross-linking.

### Senescent fibroblasts have disorganized actin cytoskeleton and upregulated integrin profile

**FIG. 4.**
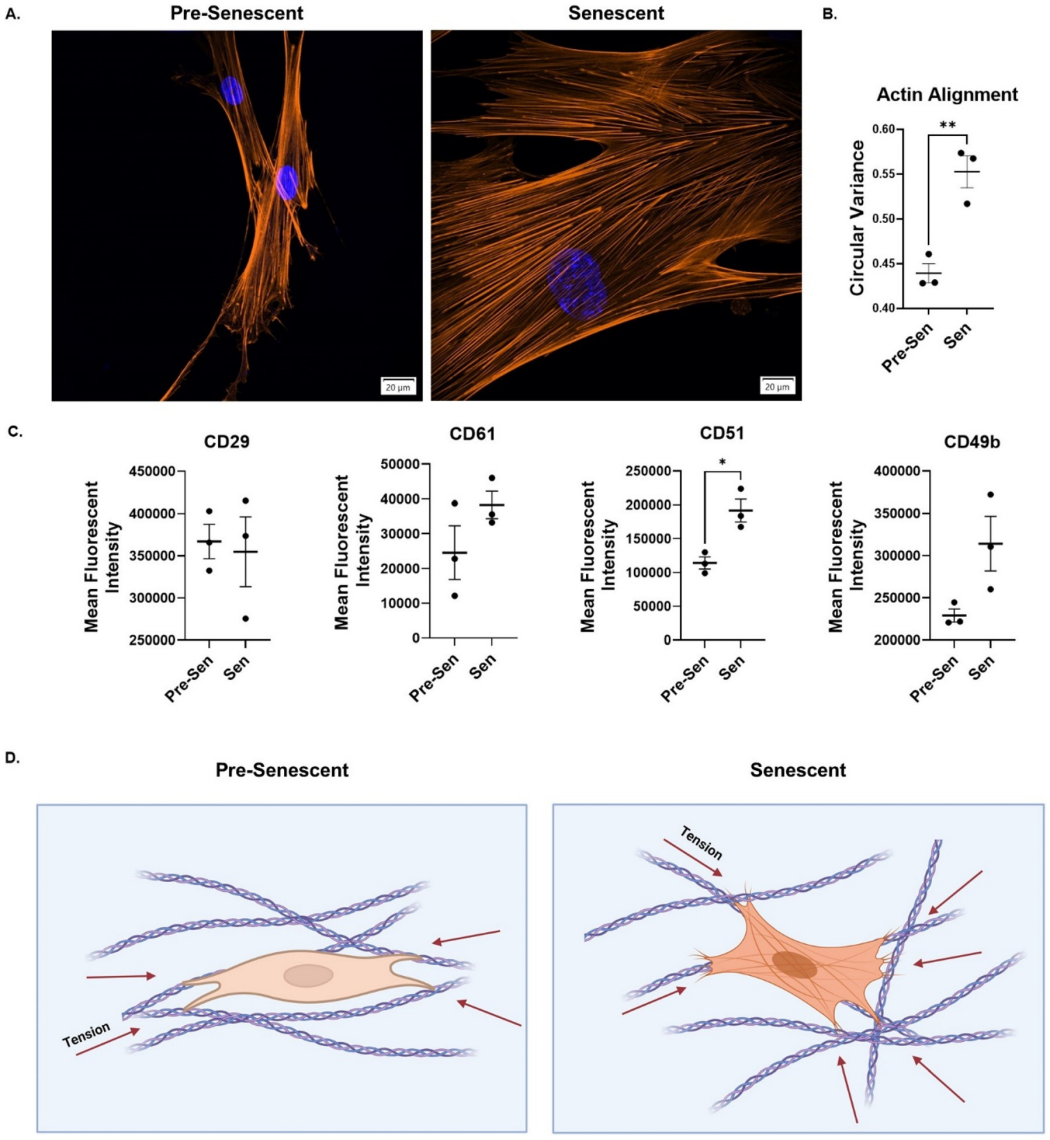
Senescent fibroblasts have disorganized actin cytoskeleton and upregulated integrin profile. (a) Representative confocal microscopy images of pre-senescent and senescent fibroblasts stained with Hoechst 33342 and phalloidin rhodamine (obj. = 60×, N.A. = 1.3, scale bar = 20 *μ*m). (b) Circular variance of actin cytoskeleton fibers for pre-senescent and senescent lung fibroblasts (n ≥ 25, N = 3). (c) Analysis of CD29, CD61, CD51, and CD49b integrin profile for pre-senescent and senescent lung fibroblasts (n ≥ 500,000 cells per condition, N = 3). (d) Schematic of proposed method of SA-ECM matrix disorganization by senescent lung fibroblasts (Created with BioRender.com). Mean ± SEM; T-Test, ^*^P ≤ 0.05, ^**^P ≤ 0.01, ^***^P ≤ 0.001, ^****^P ≤ 0.0001.

Senescent cell-matrix adhesion has previously been characterized as being significantly elevated compared to non-senescent cells, with an increased force profile, focal adhesion size, and focal adhesion kinase activity.^41,42^ ITGB3 (integrin beta 3 or β3) was also shown to regulate senescence through TGF-β signaling.^43^ Observing that senescent fibroblasts increase collagen fiber disorganization globally and locally during SA-ECM remodeling, we hypothesized that senescent fibroblasts are pulling in a multilateral fashion to promote SA-ECM disorganization. Using fluorescent imaging of actin cytoskeleton, we found that senescent fibroblasts have higher actin cytoskeleton disorganization compared to pre-senescent conditions [Figs. 4(a) and 4(b)]. Next, the abundance of CD29 (ITGB1), CD61 (ITGB3), CD51 (ITGAV), and CD49b (ITGA2) was measured using flow cytometry. CD49b and CD61 were upregulated compared to pre-senescent conditions, however, were not significantly different (P = 0.0636 and 0.1893 accordingly). CD51 was significantly upregulated compared to that of pre-senescent conditions and could be influencing matrix organization through increased cell-matrix adhesion (P = 0.0149) [Fig. 4(c)]. Upregulation of TGM2 matrix cross-linking of the lung has also been associated with pulmonary fibrosis and linked to increased fibroblast spreading and adhesion.^40,44^ We propose that SA-ECM disorganization is in part due to the increased senescent cell-matrix adhesion and multilateral contraction of the matrix due to disorganized actin cytoskeleton [Fig. 4(d)].

### Inhibition of TGFβR1 and MRTF-A induces divergent senescent pulmonary fibroblast phenotypes

**FIG. 5.**
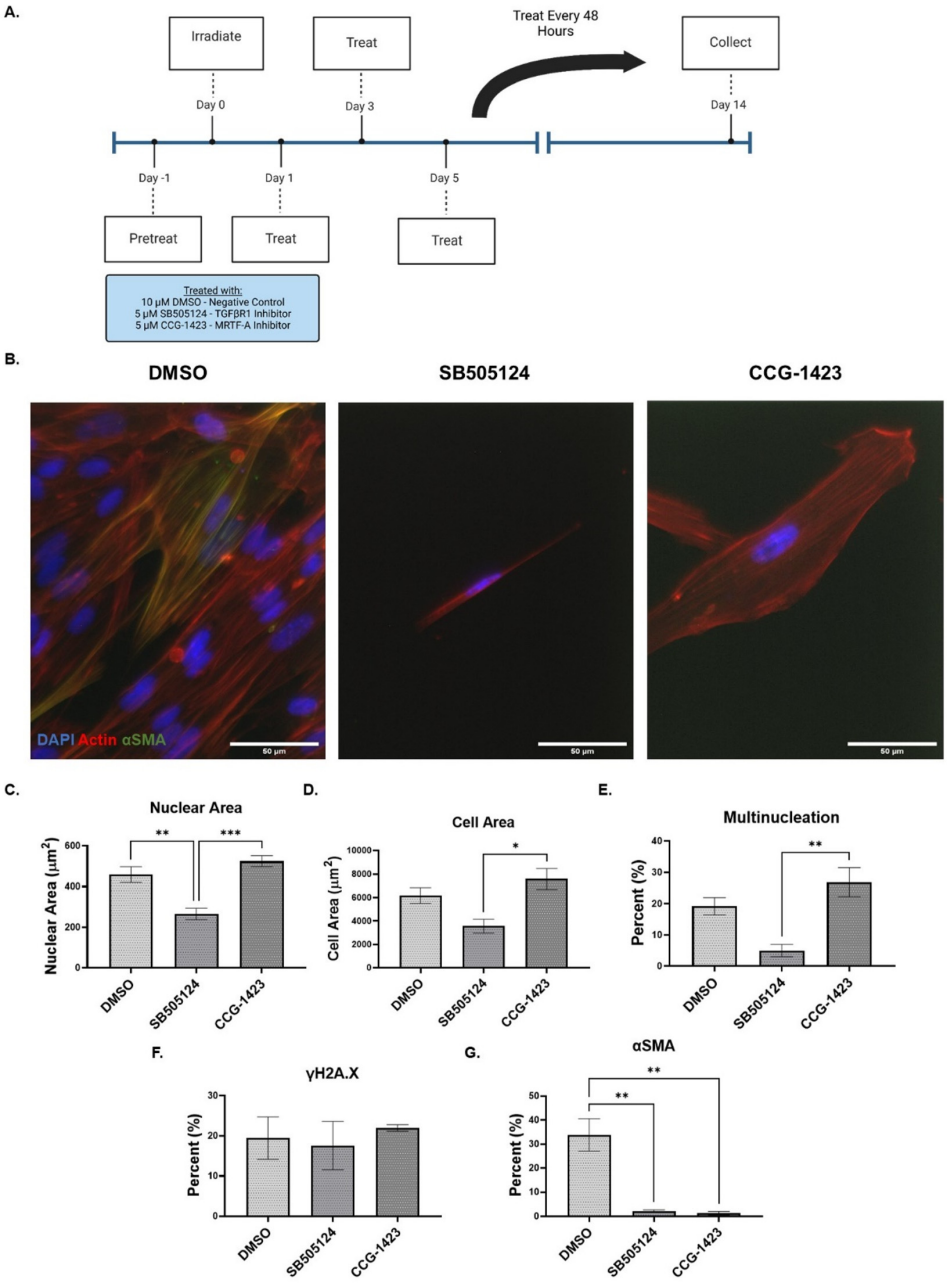
Inhibition of TGFβR1 and MRTF-A induces divergent senescent lung phenotype. (a) 24 h prior to stress-induced premature senescence is induced by γ-irradiation (15 Gy), lung fibroblasts were treated with either selective TGβFR1 inhibitor (SB505124) or MRTF-A inhibitor (CCG-1423) and cultured over 14 days (Created with BioRender.com). (b) Representative immunofluorescent images stained with Hoechst 33342 (blue), phalloidin rhodamine (red), anti-SMA (green) (obj. = 40×, N.A. = 1.3, scale bar = 50 *μ*m). (c) Inhibited senescent lung fibroblast nuclear area measured at day 14 post-irradiation (n ≥ 149, N = 3). (d) Inhibited senescent lung fibroblast cell area measured at day 14 post-irradiation (n ≥ 100, N = 3). (e) Percent of inhibited senescent fibroblasts with multinucleation (n ≥ 140, N = 3). (f) Percent of inhibited senescent fibroblasts positive for γH2A.X (n ≥ 100, N = 3). (g) Percent of inhibited senescent lung fibroblasts positive for αSMA stress-fibers (n ≥ 100, N = 3). Mean ± SEM; ANOVA. ^*^P ≤ 0.05, ^**^P ≤ 0.01, ^***^P ≤ 0.001, ^****^P ≤ 0.0001.

To further probe SA-ECM remodeling, we separately inhibited TGFβR1 and MRTF-A during senescence induction. We then characterized the senescent phenotypes over 14 days post-irradiation. TGF-β, being one of the most pro-fibrotic stimulators of ECM production and fibroblast activation, has been demonstrated to mediate paracrine senescence.^22,45–47^ MRTF-A inhibition was chosen due to its role in the mechanosensitive activation of pro-fibrotic gene expression and downstream location of TGF-β signaling processes.^30,32,48^ Using a selective inhibitor of TGFβR1 (SB505124, 5 *μ*M) and MRTF-A inhibitor (CCG-1423, 5 *μ*M) that inhibits the nuclear localization of MRTF-A, we pretreated pre-senescent fibroblasts 24 h prior to irradiation and continuously treated every 48 h post-irradiation^49–51^ [Fig. 5(a) and supplementary material Fig. 3(a)]. Senescent cell nuclear and cellular morphologies were characterized at 14 days post-irradiation using fluorescence microscopy [Fig. 5(b)]. Inhibition of TGFβR1 resulted in a decrease in nuclear and cellular area, whereas inhibition of MRTF-A resulted in an increase in nuclear and cellular area [Figs. 5(c) and 5(d)]. Interestingly, MRTF-A inhibition increased the percent of cells with multinucleation compared to negative control DMSO (19.2% vs 26.9%) and TGFβR1 inhibition decreased the percent of multinucleated cells (5%) [Fig. 5(e)]. The percent of cells expressing the DNA double strand break marker γH2A.X was not significantly different across treatment conditions [Fig. 5(f)]. Both TGFβR1 and MRTF-A influence fibroblast activation to a myofibroblast state; therefore, their inhibition reduced the percent of senescent fibroblasts expressing αSMA [Fig. 5(g)]. qRT-PCR analysis revealed TGFβR1 did result in lower expression of SASP factors, such as IL1A, IL1B, and IL6; whereby inhibiting MRTF-A increased the SASP factor expressions compared to TGFβR1 inhibition [see supplementary material Figs. 3(b)–3(d)].

### TGFβR1 inhibition reduces SA-ECM remodeling heterogeneity

**FIG. 6.**
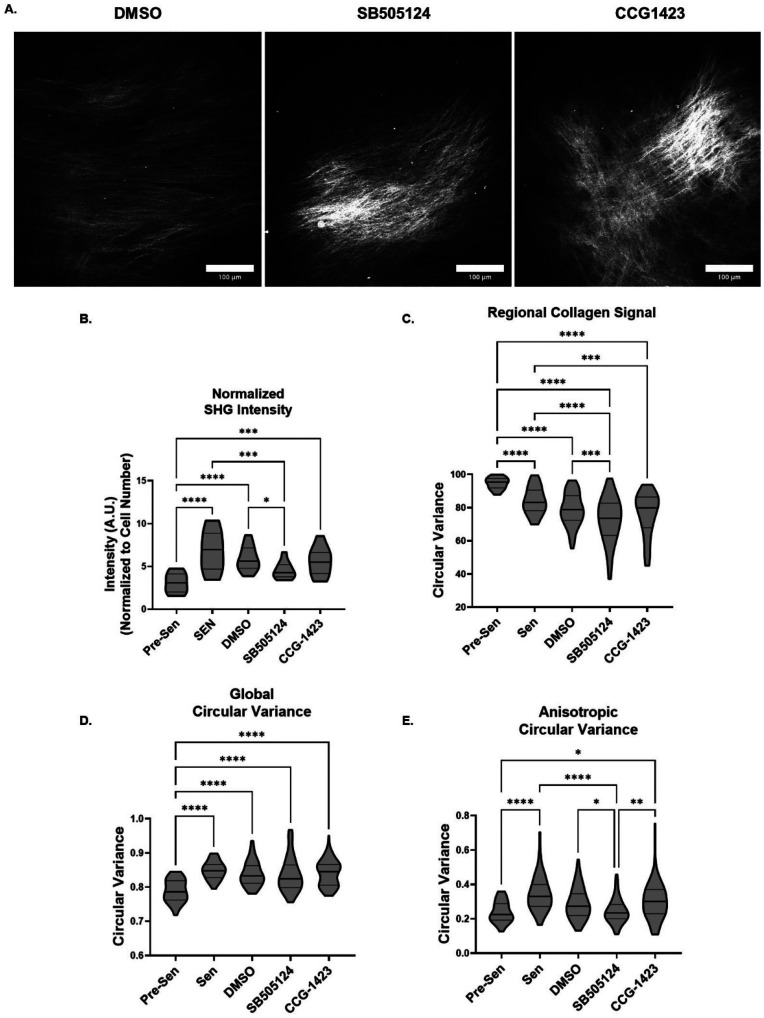
TGFβR1 inhibition reduces SA-ECM remodeling heterogeneity. (a) Representative second-harmonic generation (SHG) images of collagen fiber organization of senescent lung fibroblast-derived matrices (FDMs) treated with DMSO, SB505124, and CCG-1423 at day 14 post-irradiation (obj. = 25×, N.A. = 1.05, scale bar = 100 *μ*m). (b) Inhibited senescent lung FDM SHG intensity normalized to cell number per image (n ≥ 20, N = 3). (c) Inhibited senescent lung FDM regional collagen signal (n ≥ 34, N = 3). (D) Global circular variance of inhibited senescent lung FDM individual collagen fibers (n ≥ 34, N = 3). (e) Anisotropic circular variance of inhibited senescent lung FDM individual collagen fibers (n ≥ 34, N = 3). ANOVA; ^*^P ≤ 0.05, ^**^P ≤ 0.01, ^***^P ≤ 0.001, ^****^P ≤ 0.0001.

To observe functional differences in TGFβR1 and MRTF-A inhibited senescent fibroblast SA-ECM remodeling, inhibited senescent cells were used to grow FDMs. FDMs were treated every 48 h and isolated on day 14 to quantitatively measure SA-ECM remodeling using SHG microscopy [Fig. 6(a)]. Using this inhibition treatment plan to grow the matrices, we did not observe a difference in the average number of cells per image for the inhibited conditions [see supplementary material Fig. 4(a)]. Normalized SHG intensity for SB505124 and CCG-1423 treated conditions were not significantly different; however, TGFβR1 inhibition did significantly reduce normalized SHG intensity compared to DMSO negative control and untreated senescent conditions. It was also not significantly different from that of pre-senescent conditions [Fig. 6(b)]. Similarly, CCG-1423 and SB505124 regional collagen signals were not significantly different; however, SB505124 did significantly reduce regional collagen signal compared to pre-senescent and DMSO conditions [Fig. 6(c)]. Neither inhibitor treatments improved global circular variance of collagen fibers [Fig. 6(d)]. Interestingly, inhibition of TGFβR1 did improve local collagen fiber organization based on the significant decrease in anisotropic circular variance compared to CCG-1423, DMSO and senescent conditions. Therefore, TGFβR1 inhibition decreased the heterogeneity in collagen fiber organization back to pre-senescent condition levels [Fig. 6(e)].

### Human pulmonary interstitial fibrosis tissue samples increase in collagen fiber disorganization with age and p16 expression

**FIG. 7.**
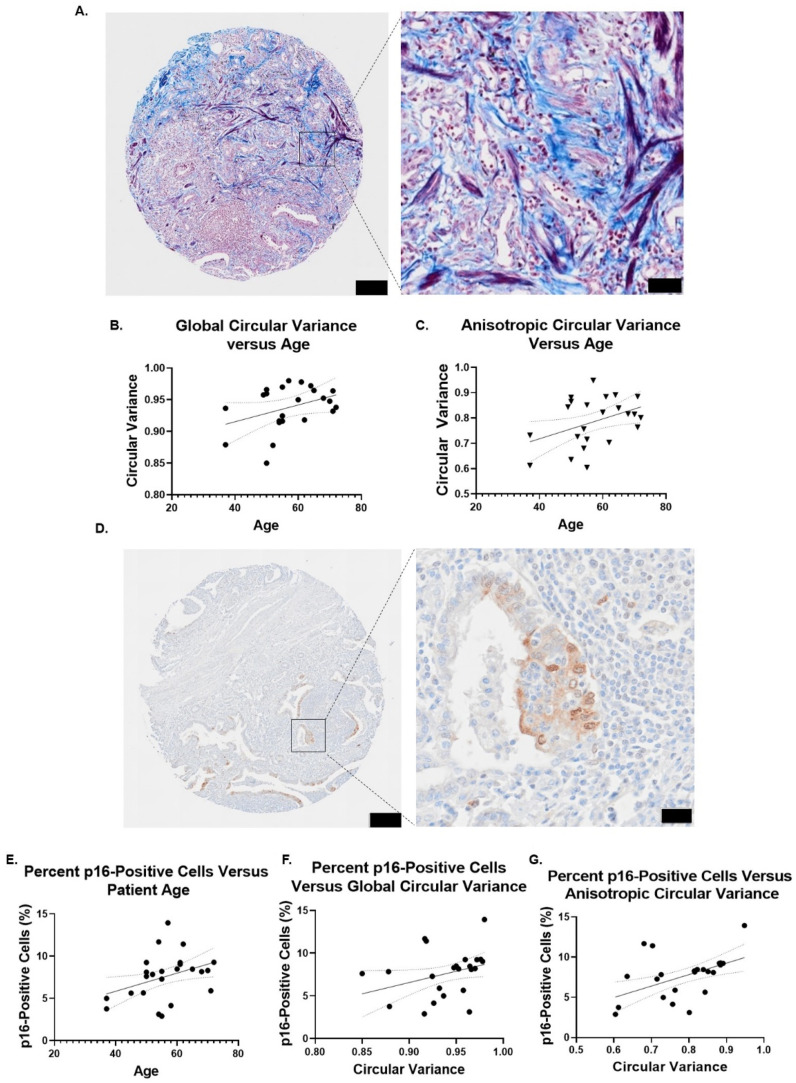
Regional matrix analysis of human interstitial pulmonary fibrosis tissue samples. (a) Representative image of Masson's trichrome stained interstitial (obj. = 40×, N.A. = 0.95, large image scale bar = 200 *μ*m, zoom scale bar = 50 *μ*m). (b) Simple linear regression of global circular variance vs age of pulmonary fibrosis patient (n = 2 samples per patient, N = 23 patients). (c) Simple linear regression of anisotropic circular variance vs age of pulmonary fibrosis patient (n = 2 samples per patient, N = 23 patients). (d) Representative image of p16-stained pulmonary fibrosis patient tissue sample (obj. = 40×, N.A. = 0.95, large image scale bar = 200 *μ*m, zoom scale bar = 20 *μ*m). (e) Simple linear regression of percent of p16-positive cells vs age of pulmonary fibrosis patient (n = 2 samples per patient, N = 23 patients). (f) Simple linear regression of percent of p16-positive cells vs global circular variance of pulmonary fibrosis patient (n = 2 samples per patient, N = 23 patients). (g) Simple linear regression of percent of p16-positive cells vs anisotropic circular variance of pulmonary fibrosis patient (n = 2 samples per patient, N = 23 patients). Simple linear regression. ^*^P ≤ 0.05, ^**^P ≤ 0.01, ^***^P ≤ 0.001, ^****^P ≤ 0.0001.

Having quantified that senescent lung fibroblast SA-ECM remodeling establishes a disordered collagen architecture and with prior studies indicating that senescent cells play an intricate role in the development of pulmonary fibrosis, we examined the clinical relation between senescent cells and collagen fiber disorganization in human pulmonary fibrosis patient samples. Human interstitial pulmonary fibrosis patient tissue microarrays stained with Masson's trichrome ranged in age from 37 to 72 years (n = 2 samples per patient, N = 23 patients) [Fig. 7(a)]. Using color deconvolution to extract stained collagen fiber regions, the same regional collagen fiber analysis used to analyze individual fiber organization for the senescent FDMs was applied to the stained patient tissue [see supplementary material Fig. 5(a)]. Patient samples were binned by age according to the Freedman–Diaconis rule to examine if there were any changes in the percent of regions with no collagen (dark) and anisotropic (organized) and isotropic (disorganized) collagen fibers. Patient samples did not vary significantly in the proportion of regions identified based on age [see supplementary material Fig. 5(b)]. A simple linear regression was used to examine if there were linear trends between global circular variance and anisotropic circular variance vs age. No trend was found between age and global circular variance (P = 0.0874, r^2^ = 0.076 90) [Fig. 7(b)]. However, a significant trend was observed between anisotropic circular variance and age, indicating a linear relationship between age and local collagen fiber disorder (P = 0.0311, r^2^ = 0.1110) [Fig. 7(c)]. Having observed a trend between age and local collagen fiber disorganization, a second tissue microarray consisting of the same human interstitial pulmonary fibrosis patients was stained for the senescence marker p16 (n = 2 samples per patient, N = 23 patients) [Fig. 7(d)]. We identified an age-dependent increase in the percent of p16-positive cells within the tissue (P = 0.0247, R^2^ = 0.1119) [Fig. 7(e)]. Combining the p16-positive staining and Masson's trichrome analysis per patient, we then used a simple linear regression to investigate the relationship between the percent of p16-positive cells and matrix organization parameters. Herein, we did not observe a significant relationship between global circular variance and the percent of p16-positive cells identified (P = 0.065, R^2^ = 0.08063) [Fig. 7(f)]. Nonetheless, we did measure a significant positive correlation (Pearson r = 0.4705, P = 0.0235) when comparing the percent of p16-positive cells and anisotropic circular variance. This resulted in a significant linear trend based on a simple linear regression, demonstrating that as the percent of p16-positive cells increased, local collagen fiber disorganization also did (P = 0.0029, R^2^ = 0.1966) [Fig. 7(g)].

## CONCLUSIONS

In this study, we comprehensively mapped the spatiotemporal dynamics of SA-ECM remodeling by designing an *in vitro* senescent lung fibroblast-derived matrix (FDM) model to further understand the previously ambiguous role of SA-ECM remodeling in age-related disease development. Applying multiphoton SHG microscopy, we observed a temporally heterogeneous biphasic SA-ECM remodeling process that first underwent matrix degradation followed by an increase in matrix density. Like prior findings, we found that ECM-related gene expression decreased over time, while MMP activity increased.^14^ We believe that healthy soluble collagen is initially degraded through SASP, and an increase in matrix density during the second phase of SA-ECM remodeling is due to the accumulation of overly modified insoluble matrix rather than an increase in matrix deposition by senescent fibroblasts.

Although at an early timepoint, senescent and pre-senescent matrix architectures were not significantly different, the resulting stabilized senescent matrix was significantly disorganized compared to pre-senescent lung fibroblast ECM architectures. It has been shown that the senescent cell phenotype is hyper-adhesive, with increased mechanical tension that can regulate gene expression through integrin binding.^41–43^ However, it was previously unclear whether senescent fibroblasts biophysically influenced the surrounding local matrix organization during senescent matrix development.^52^ Our study demonstrated that senescent fibroblasts do in fact promote matrix disorganization and we propose that fibroblast SA-ECM disorder is, in part, the result of multilateral tension applied to the matrix through disordered actin cytoskeleton and heightened matrix adhesion. Having observed a biophysical difference between senescent and pre-senescent fibroblasts, we sought to inhibit the biomechanical contributions of SA-ECM remodeling through MRTF-A during senescent induction. Profibrotic cytokine TGF-β was also previously shown to mediate paracrine senescence and SASP; therefore, we also inhibited TGF-β signaling through TGFβR1 during senescence induction.^22^ Doing so, we generated divergent senescent fibroblast phenotypes to grow senescent lung FDMs. We found that inhibition of TGFβR1 improved SA-ECM matrix organization.

Cellular senescence has previously been shown to mediate pulmonary fibrosis development and progression.^8,18,27^ Having established that SA-ECM remodeling varies temporally to establish a stable disorganized senescent architecture, we hypothesized that ECM architecture of patients diagnosed with interstitial pulmonary fibrosis would vary depending on age. Using Masson's trichrome staining and color deconvolution, the same ECM regional analysis approach applied to the senescent lung FDM model was utilized to characterize patient sample matrix architecture. Doing so, we identified that as patient age increases, so does local matrix disorganization. Having observed this age and matrix disorganization relationship, we stained tissue samples from the same patients for the senescence marker p16. Furthermore, increased percent of p16-positive cells were positively correlated with an increase in local collagen fiber disorder. These findings suggest that local loss in structural organization is related to age and the senescence marker p16. Moreover, this loss in structure during disease development could further contribute to the decline in tissue function. Likewise, the age-dependent decline in lung function has been shown to be directly related to pulmonary ECM remodeling and the increase in cellular senescence within tissue.^53,54^

Through the design, development, and spatiotemporal monitoring of *in vitro* senescent lung FDMs, our study further provided insights into the understudied topic of SA-ECM remodeling. The senescent fibroblast phenotype undergoes a parabolic-like remodeling process that establishes a heterogeneously disorganized SA-ECM architecture. We also demonstrated that local SA-ECM collagen fiber disorder can be therapeutically targeted and improved. The results of this study offer further untapped potential for the utilization of senescent fibroblast matrix as an aged biomaterial for the development of 3D age-related disease models in the future. By collecting and reconstituting decellularized SA-ECM into a 3D hydrogel, 3D bioprinting could be used to design aging ECM architectures. 3D hydrogels made from reconstituted SA-ECM would further allow for the investigation of the influence of senescent cell matrix on age-related disease, senescent cell biology, cancer development, and therapeutic development.^55^ This study unveils new insights into senescent cell ECM remodeling by spatially and temporally quantifying SA-ECM dynamics. In doing so, we demonstrate that senescent cells *in vitro* and *ex vivo* play an intricate role in the disorganization of the matrix architecture.

## METHODS

### Cell culture

The primary human lung fibroblast cell line, LF1 (a gift from Dr. John Sedivy, Brown University, Providence, RI), was previously described.^56,57^ Fibroblasts were cultured in Dulbecco's Modified Eagle's Medium F-12 (DMEM-F12) (Corning) supplemented with 10% fetal bovine serum (FBS) (Atlanta Biologicals), 15 mM HEPES (Fisher Bioreagents), 1% L-Glutamine (Cytivia), and 1% penicillin-streptomycin (P/S) (Corning). All cells were maintained at 37 °C, 5% CO_2_, and 2% oxygen.

### Irradiation

LF1s were cultured to 80% confluence and exposed to 15 Gy γ-irradiation using a Mark I 68A137CS Irradiator at a rate of 5.04 Gy/min for 3 min. For the generation of FDMs, irradiated cells were immediately transferred to gelatin-coated coverslips. This mode of senescence induction was selected to generate large populations of senescent lung fibroblasts for the direct comparison of pre-senescent and senescent conditions.

### Fibroblast-derived matrices

Fibroblast-derived matrices were prepared using a modified version of the following protocol.^58^ Within a 12-well plate (Corning), 15 mm diameter, 1.5-*μ*m-thick glass coverslips (Electron Microscopy Sciences) were coated with sterile 0.2% gelatin (Fisher Scientific) and incubated at 37 °C for 1 h before fixation with 1% glutaraldehyde (Electron Microscopy Sciences) for 30 min. Post-fixation, coverslips were washed using phosphate buffered saline (PBS) (Corning). 1M ethanolamine (Acros Organics) was added to each coverslip for 30 min and subsequently washed using PBS. Senescent and pre-senescent LF1 cells were then seeded at 80% confluence to each prepared coverslip. LF1 cells were maintained using matrix media consisting of DMEM-F12, 10% fetal bovine serum (FBS), 15 mM HEPES, 1% P/S, 50 *μ*g/ml L-ascorbic acid salt (Acros Organics), and 60 *μ*g/ml L-Proline (Alfa Aesar). Media was replaced every 48 h throughout experimentation.

### Scanning electron microscopy

Pre-senescent and senescent fibroblast-derived matrices were cultured for 14 days. Matrices were decellularized using 20 mM ammonium hydroxide and 0.5% Triton-X in DI water for 2 h. Matrices were then washed with PBS 3 times for 30 min and a final wash in PBS overnight. Matrices were fixed in Karnovsky's fixative (Electron Microscopy Sciences, #15720) overnight. Fixed matrices were then washed 5 times for 3 min in cold 0.15 M sodium cacodylate buffer (Electron Microscopy Sciences, #11654). Matrices were sequentially dehydrated in 25%, 50%, 75%, and 100% ethanol (fresh) for 15 min each. Samples were critical point dried with liquid CO_2_ and sputter coated with 100 Å gold palladium and visualized with a Thermo Fisher Scientific Apreo VS scanning electron microscope.

### Multiphoton second-harmonic generation and single photon imaging

To generate and detect second-harmonic signal, an Olympus FV-10000MPE multiphoton microscope (Olympus, Tokyo, Japan) fitted with a 25× water-immersion objective (NA 1.05, WD 2 mm) and second-harmonics bandpass filter (405/40) was used. The excitation wavelength was set to 800 nm using a Mai Tai HP tunable laser. Samples were fixed with 4% formaldehyde, permeabilized using 0.5% Triton X-100, and stained with RedDot2^TM^ far-red nuclear stain (1:200, Biotium, #40061). Single-photon fluorescence imaging was done using the same setup listed earlier. Images were exported as OIB (Olympus Image Binary) and converted to tiffs using ImageJ. Quantitative analysis was done through an in-house MATLAB code previously published in Refs. 23, 24, and 26.

### 2D Fourier transform second-harmonic generation microscopy image analysis

Using an in-house MATLAB code developed in the Toussaint Lab previously described in the literature, 2D fast Fourier transform (2D-FFT) analysis was combined with SHG microscopy to quantify spatial collagen fiber organization of lung fibroblast-derived matrices.^24,25^ A 16 × 16-pixel^2^ grid array was then overlaid on SHG images and within each 16  × 16-pixel^2^ grid region, the individual collagen fiber orientations were calculated and averaged. These regions were classified as anisotropic (organized, collagen fibers have a preferred orientation), isotropic (disorganized, collagen fibers do not have a preferred orientation), and dark (no signal) based on circular variance. In each region of the image, the orientation of the collagen fibers was calculated based on the gradient of pixel intensities. The orientation is then represented as a normal resultant vector. The normalized resultant vectors for each region were then averaged to get a mean resultant vector (R). Circular variance (CV) is then calculated as one minus the length of the mean resultant vector,

CV=1−R.

### Proliferation

1 mg/ml bromodeoxyuridine/5-bromo-2′-deoxyuridine (BrdU) (MilliporeSigma) in cell culture media was used to treat LF1 cells seeded on 12-mm-diameter coverslips. After a 24-h incubation, cells were fixed using 4% formaldehyde and permeabilized using 0.5% Triton X-100 in PBS. Cells were then treated with 3M HCL and blocked with 5% horse serum in PBS. Cells were then incubated in biotinylated anti-BrdU primary antibody (1:100, BioLegend, No. 339810) followed by secondary antibody DyLight^TM^ 488 Streptavidin (1:200, BioLegend, cat. No.405218) and propidium iodide (PI). Coverslips were mounted onto glass slides using Fluoromount-G (SouthernBiotech, Cat. No. 0100-01) and imaged using a Nikon Eclipse TI inverted microscope at 10x magnification. LF1 proliferation was quantified based on the number of BrdU-positive cells normalized to the total number of cells using ImageJ.

### Immunofluorescence staining

Within a 24-well plate (Corning), cells seeded on 12 mm-diameter, #1.5 glass coverslips were fixed using 4% paraformaldehyde. Following this, cells were permeabilized using 0.5% Triton X-100. Next, coverslips were incubated overnight in anti-αSMA (1:200, Cell Signaling, #19245) and anti-γH2A.X (1:100, Cell Signaling, #80312) primary antibodies. Goat anti-rabbit Alexa Fluor 488 secondary antibody (1:1000 dilution, Invitrogen, A32731) and goat anti-mouse Alexa Fluor 647 secondary antibody (1:500, SouthernBiotech, Cat. No.1030-31) were used in conjunction with Alexa Fluor 555 Phalloidin (1:400, Thermo Fisher Scientific, A34055), and Hoechst 33342 (1:2000, Thermo Fisher Scientific, #62249) for 1 h. The coverslips were washed with PBS and then mounted on to glass slides using Fluoromount-G (SouthernBiotech, Cat. No. 0100-01) and imaged using a Nikon Eclipse TI inverted at 10× and 40× magnification. Nuclear areas and nuclear shape factors were processed through a custom pipeline in CellProfiler (Broad Institute). Images were thresholded and segmented using the IdentifyPrimaryObjects module. Cellular area and cell form factor measurements were manually done through tracing with ImageJ. Percent positivity of α-SMA and γH2A.X was quantified based on the number of positive cells normalized to the total number of cells.

### Real-time PCR

All reagents were purchased from Bio-Rad for the RNA isolation, cDNA synthesis, and qRT-PCR. Total mRNA was extracted from the senescent and pre-senescent fibroblasts using Ribozol^TM^ RNA Extraction Reagent (VWR Life Science, Cat. No. N580-100ML) according to the manufacturer's instructions. RNA purity and integrity were measured using a Nanodrop 1000 and iScript gDNA clear cDNA synthesis kit (Bio-Rad, Cat. No. 1725035) was used for cDNA synthesis. 1 *μ*g of RNA was used to generate cDNA and conduct qRT-PCR using a CFX Connect Real-Time System (Bio-Rad) using the following primers:

P16, (Sense) 5′-CACTCACGCCCTAAGC-3′ and (antisense) 5′-GCAGTGTGACTCAAGAGAA-3′; P21, (sense) 5′-CGATGGAACTTCGACTTTGTCA-3′ and (antisense) 5′-GCACAAGGGTACAAGACAGTG-3′; COL1A1, (sense) 5′-ATCAACCGGAGGAATTTCCGT-3′ and (antisense) 5′-CACCAGGACGACCAGGTTTTC-3′; COL3A1, (sense) 5′-GGAGCTGGCTACTTCTCGC-3′ and (antisense) 5′-GGGAACATCCTCCTTCAACAG-3′; TGM2, (sense) 5′-GAGGAGCTGGTCTTAGAGAGG-3′ and (antisense) 5′ CGGTCACGACACTGAAGGTG3′; LOX, (sense) 5′-CGGCGGAGGAAAACTGTCT-3′ and (antisense) 5′-TCGGCTGGGTAAGAAATCTGA-3′; IL1A, (sense) 5′-TGGTAGTAGCAACCAACGGGA-3′ and (antisense) 5′-ACTTTGATTGAGGGCGTCATTC-3′; IL1B, (sense) 5′-TGCACGCTCCGGGACTCACA-3′ and (antisense) 5′-CATGGAGAACACCACTTGTTGCTCC-3′; IL6, (sense) 5′-ACTCACCTCTTCAGAACGAATTG-3′ and (antisense) 5′-CCATCTTTGGAAGGTTCAGGTTG-3′; β-Actin, (sense) 5′-AGAGCTACGAGCTGCCTGAC-3′ and (antisense) 5′-AGCACTGTGTTGGCGTACAG.

β-actin was used as the housekeeping gene and the pre-senescent condition for each timepoint was used as the control group for normalization.

### Confocal microscopy

Pre-senescent and senescent LF1s were cultured for 14 days and fixed in 2% paraformaldehyde. Cells were permeabilized using 0.5% Triton X-100 and stained with Hoechst 33342 (1:2000, Thermo Fisher Scientific, #62249) and Alexa Fluor 555 Phalloidin (1:400, Thermo Fisher Scientific, A34055). Spinning disk confocal microscopy was used to image actin cytoskeleton alignment (Olympus IX83 inverted microscope) (silicone oil immersion obj. = 60×, N.A. = 1.3). Confocal microscope images were uploaded to ImageJ (FIJI) and channels were separated. Actin fiber circular variance was extracted from tiff images containing individual cells using an in-house 2D fast Fourier transform (2D-FFT) MATLAB code.^37,38^ The MATLAB code overlaid a grid system, consisting of 16 × 16-pixel^2^ regions. In each region of the image, the orientation of actin fibers was calculated based on the gradient of pixel intensities. This orientation was then represented as a normal resultant vector. The normalized resultant vectors for each region were then averaged to get a mean resultant vector (R). Circular variance (CV) of the actin fibers was then calculated as one minus the length of the mean resultant vector. There were no changes to the MATLAB analysis pipeline when analyzing the FDMs or the cell actin alignment.

### FDM DQ-gelatin proteolytic activity

Pre-senescent and senescent FDMs were treated with 100 *μ*g/ml of DQ gelatin in media and incubated at 37 °C for 2 h. Samples were fixed with 4% formaldehyde, permeabilized with 0.5% Triton X-100, and stained with RedDot2^TM^ far-red nuclear stain (1:200, Biotium, #40061). Images were exported as OIB (Olympus Image Binary) and converted to tiffs using ImageJ. DQ gelatin fluorescence intensity was quantified using ImageJ and normalized using the cell number per image.

### MTT assay

Senescent pulmonary fibroblasts were seeded into 96-well plates and treated with either culture media, DMSO, 1, 5, and 10 *μ*M SB505124 (Cayman Chemical, No. 11793) or CCG-1423 (Cayman Chemical, No. 10010350) every 48 h for 14 days. Samples were incubated in MTT solution for 3 h at 37 °C before MTT solvent was added. Samples were incubated in MTT solvent for 30 min at 37 °C prior to absorbance reading at 590 nm with a reference filter of 620 nm. Sample MTT absorbances were normalized to the culture media condition for comparison.

### Flow cytometry

Pre-senescent and senescent LF1s were cultured for 14 days. Cells were detached, pelleted, and suspended in FACS buffer (PBS, 2% FBS, 1 mM EDTA), and fixed in 2% PFA. Cells were stained for the surface markers anti-human CD29 (BioLegend #303003), anti-human CD49b (BioLegend #359309), anti-human CD51 (BioLegend #327907), or anti-human CD61 (BioLegend #336405). A total of 500 000 cells per condition were analyzed. Samples were run on a Cytek Aurora flow cytometer.

### Tissue microarray histology analysis

Unstained human pulmonary interstitial fibrosis tissue microarrays were purchased from US Biomax, Inc. (LC561). Tissue microarrays were stained with Masson's trichrome and for p16 by trained pathologist Dr. Yihong Wang. Images were taken using an Olympus VS200 Slide Scanner (obj. 40×, N.A. = 0.95). Masson's trichrome images were color deconvoluted using ImageJ to extract only the ECM component of the sample. Using the same in-house 2D-FFT MATLAB pipeline previously used, regional collagen fiber analysis was conducted. Tissue microarrays stained for p16 were pre-processed prior to analysis using ImageJ. Hue and saturation thresholding were consistently applied across all images to remove carbon-laden regions. Next, a brightness threshold was set using ImageJ to enable the specific identification of the carbon-laden cells [see supplementary material Fig. 5(c)]. A white mask was superimposed over the carbon cell regions leaving the p16 stained cells unaltered [see supplementary material Fig. 5(d)]. CellProfiler was then used to analyze the ImageJ pre-processed p16 stained tissue samples. A simple linear regression analysis was conducted to determine data trends. For the analysis of histology, no adjustments were made to the MATLAB circular variance analysis pipeline.

### Statistical analysis

Prism software was used for statistical analysis. Results are reported as mean ± SEM or in violin plots. For the comparison of multiple groups, one-way ANOVA was used with Tukey's method for statistical hypothesis testing. For comparisons between only two groups, Student's t-test was utilized. To examine clinical patient data, a simple linear regression was used. ^*^P *<* 0.05 was considered significant. For consistency, significance values were as follows: ^*^P *<* 0.05, ^**^P *<* 0.01, ^***^P *<* 0.001, and ^****^P *<* 0.0001.

## SUPPLEMENTARY MATERIAL

See the supplementary material for Fig. 1: αSMA and γH2A.X characterization; Fig. 3: Pre-senescent and senescent lung fibroblast cell count, proteolytic activity, and ECM remodeling gene regulation; Fig. 5: Inhibitor dose-dependent lung fibroblast viability and interleukin gene regulation changes; Fig. 6: Inhibitor treated average senescent cell number per image; and Fig. 7: Masson's trichrome collagen fiber extraction analysis and p16 sample carbon-laden regions.

## Data Availability

The data that support the findings of this study are available from the corresponding author upon reasonable request.
